# Quality of life of Brazilian and Spanish cancer patients undergoing chemotherapy: an integrative literature review

**DOI:** 10.1590/1518-8345.0564.2688

**Published:** 2016-05-17

**Authors:** Namie Okino Sawada, Adriana Cristina Nicolussi, Juliana Maria de Paula, Maria Paz Garcia-Caro, Celia Marti-Garcia, Francisco Cruz-Quintana

**Affiliations:** 1 PhD, Associate Professor, Escola de Enfermagem de Ribeirão Preto, Universidade de São Paulo, PAHO/WHO Collaborating Centre for Nursing Research Development, Ribeirão Preto, SP, Brazil; 2 RN, PhD, Hospital das Clínicas de Ribeirão Preto, Faculdade de Medicina de Ribeirão Preto, Universidade de São Paulo, SP, Brazil; 3 Doctoral Student, Escola de Enfermagem de Ribeirão Preto, Universidade de São Paulo, PAHO/WHO Collaborating Centre for Nursing Research Development, Ribeirão Preto, SP, Brazil; 4 PhD, Assistant Professor, Departamento de Enfermagem, Universidade de Granada, Granada, Andaluzia, Spain; 5 PhD, Full Professor, Departamento de Enfermagem, Universidade de Granada, Granada, Andaluzia, Spain

**Keywords:** Quality of Life, Neoplasms, Drug Therapy, Review

## Abstract

**Objective:**

characterize the scientific production of Brazil and Spain in regard to methodological aspects and aspects of health-related quality of life experienced by cancer patients receiving chemotherapy in both countries.

**Method:**

integrative literature review was conducted using the following databases: CINAHL, MEDLINE, SCOPUS and CUIDEN and the electronic libraries PubMed and SciELO, conducted in September 2013.

**Results:**

a total of 28 papers met the inclusion criteria. The synthesis of knowledge was presented in three categories of analysis: assessment of quality of life in different types of cancer; sociodemographic factors that influenced quality of life; and type of cancer and interventions that improve quality of life. Chemotherapy affects health-related quality of life and the most important factors were: age, sex, chemotherapy protocol, type of surgery, stage of the disease, educational level, and emotional intelligence. Complementary therapies such as acupuncture, guided visualization, prayers and exercise were positive and reduced side effects.

**Conclusion:**

the results showed a poor level of evidence, since 86% of the studies were cross-sectional descriptive studies; the instrument most frequently used to measure health-related quality of life was EORTC QLQ C-30 and more studies were conducted in Brazil than in Spain.

## Introduction

The number of new cases of cancer, a disease relevant to public health, has increased in Brazil and Spain. The incidence and prevalence of cancer, as well as cancer mortality rates, are similar in Brazil to those found in Spain, as are health policies established in the oncological field. The objective of the Brazilian National Oncological Care Policy, established by Decree No. 2.439/GM on December 8, 2005^(^ 1 ^)^ and the Spanish Cancer Strategy of the National System of Health, Ministry of Health and Social Policy (2010)^(^ 2 ^)^, is to optimize the prevention, diagnosis, and treatment of cancer, as well as to improve information and encourage scientific investigation. Lines of action also converge, emphasizing health promotion, prevention, diagnosis, treatment, rehabilitation, palliative care, quality of life, and research.

The surgical treatment of cancer and other therapeutic procedures (radiotherapy and chemotherapy) has reduced mortality and morbidity; however, there is a concern regarding the functional status and quality of life of these individuals. 

Hence, the rehabilitation of patients with cancer is a continuous process, the purpose of which is to maximize the capacities of these individuals within the limitations imposed by the disease and treatment. In this context, we stress the need to investigate the health-related quality of life of both Brazilian and Spanish cancer patients treated with chemotherapy.

Even though, up to the present, studies have not reached a consensus in regard to the concept of Quality of Life (QoL), three aspects are common in all the definitions, namely: subjectivity, dimensionality, and bipolarity. As shown in the Health-Related Quality of Life (HRQL) review, the individuals' perceived health status is often used. This perception refers to the extent a chronic disease or condition, in addition to its symptoms, interferes in individuals' daily lives^(^ 3 ^)^.

Given the previous discussion, this Integrative Review's aim was to investigate what the literature has produced in regard to the Health-Related Quality of Life of cancer patients undergoing chemotherapy in Brazil and in Spain, seeking evidence to support the discussion of a larger project that compares QoL of cancer patients undergoing chemotherapy in Brazil and Spain and then critically analyze the results to obtain relevant information to integrate evidence with healthcare practice.

The specific objectives of this IR include: characterize scientific publications in Brazil and Spain in regard to methodological characteristics; characterize studies in regard to aspects of HRQL presented by cancer patients treated with chemotherapy in both countries; identify the HRQL domains that are affected in this population, in addition to sociodemographic and clinical factors that affect HRQL; and identify what instruments are used to assess HRQL in these studies. 

## Method

This is an Integrative Literature Review (IR), considered a strategy to identify existing evidence to ground healthcare practices. The IR is a method that enables the search for, clinical assessment of, and synthesis of evidence available on the topic under study^(^ 4 ^)^. IR enables the inclusion of diverse methodologies (experimental and non-experimental studies) and contributes to the presentation of varied perspectives regarding a phenomenon and is a means to integrate scientific knowledge in a certain field, contributing to clinical practice. 

Hence, this review was conducted according to a six stage method: select the hypothesis or review question; select the sample; define the study's characteristics; analyze the studies included in the review; interpret the results; and present the review with a synthesis of knowledge^(^ 4 ^)^ **_._**

The first guiding question was: "what is the knowledge produced concerning the HRQL of Brazilian and Spanish cancer patients undergoing chemotherapy?".

In the second stage, it is important to characterize the sample with inclusion and exclusion criteria. For that, the following databases were used: CINAHL, MEDLINE, SCOPUS and CUIDEN and the electronic libraries PubMed and SciELO, using the platforms EBSCOhost, ProQUEST and the Index Foundation to search the CUIDEN database. These databases were chosen because they are very comprehensive, while SciELO and CUIDEN were chosen because they include papers addressing Brazilian and Spanish patients. SciELO was created in 2002 and gathers a large collection of scientific Brazilian periodicals and the CUIDEN database was created in 1991, under the auspices of the CEDEC (Nursing Community Documentation Center) and, later, of the Index Foundation, to improve the dissemination of scientific knowledge specializing in health care in Spain^(^ 5 ^)^.

The descriptors/key words used in the four databases and two electronic libraries were: quality of life, health-related quality of life, cancer, and chemotherapy; the Boolean operators "and" and "or" were used.

Inclusion criteria were: studies addressing the QoL of Brazilian and Spanish cancer patients undergoing chemotherapy, with a sample of Brazilian or Spanish patients 18 years old or older, written in Portuguese, English or Spanish, indexed in the CINAHL, MEDLINE, SCOPUS or CUIDEN databases or in the PubMed and SciELO electronic libraries, published in the last five years (from January 2009 to September 2013), whose abstracts were available.

Exclusion criteria were: theses and dissertations, papers related to methodological research such as the creation and/or validation of QoL instruments, multi-center studies, whose exclusive population was not Brazilian or Spanish, systematic reviews, and those not including human beings. 

The same instrument used in a previous study^(^ 6 ^)^ was adopted in the third stage that consists of the definition of the study's characteristics. Three experts assessed the instrument's apparent and content validity. It contains the identification of studies (periodical, authors' background and affiliation, language, and country of origin), methodological aspects (study design, objectives, sampling, data treatment, results, conclusions, and level of evidence) and characteristics of QoL (definition of QoL, instruments and domains). The item related to the characteristics of ostomy according to the chemotherapy protocol, one of this review's points of interest, was changed. 

For level of evidence, the following classification was considered: level 1 evidence accruing from systematic reviews or meta-analyses of Controlled Randomized Clinical Trials (CRCT); level 2 refers to evidence obtained from at least one well-delineated CRCT; level 3 refers to evidence from well-delineated non-randomized clinical trials; level 4 originates from well-delineated case-control and cohort studies; level 5 refers to systematic reviews of descriptive or qualitative studies; level 6 refers to evidence originating from a single descriptive or qualitative study; and level 7 refers to evidence originating from the opinion of authorities and/or expert committees^(7).^

A detailed analysis of papers was conducted in the fourth stage, paying attention to inclusion and exclusion items, while a deepened analysis was conducted in the fifth stage in order to achieve the study's objectives and compare data found in the literature. The sixth stage was the final one, in which a synthesis of the knowledge acquired in the review was presented through thematic categories, tables and figures.

## Results

The search for papers in the CINAHL, MEDLINE, EBSCO and CUIDEN databases and SciELO and PubMed electronic libraries was conducted in September 2013 using the keywords: quality of life, health-related quality of life, cancer, and chemotherapy, with a combination of two or three words.

Table 1 shows the quantity of papers that were found, those found in more than one database, and those selected according to database.

**Table 1:** - Number of papers obtained from databases. Brazil/Spain, 2009-2013

Databases	Papers found	Papers excluded	Papers selected	Papers that appeared more than once	Total papers analyzed
MEDLINE	330	322	08	04	08
CINAHL	489	478	11	10	09
SCOPUS	218	201	17	09	08
SciELO	45	33	12	09	03
PubMed	100	100	0	0	0
CUIDEN	03	03	0	0	0
Total	1185	1137	48	32	28

Inclusion criteria, i.e., concerning Brazilian or Spanish patients 18 years old or older, papers published in the last five years (January 2009 to October 2013), written in Portuguese, English or Spanish, addressing human subjects, were applied in the search of the databases and a total of 1,185 papers were identified. After reading the abstract, a total of 48 full-text papers were selected, of which 32 appeared more than once; only 28 met the inclusion criteria and composed the IR.

**Figure 1:**
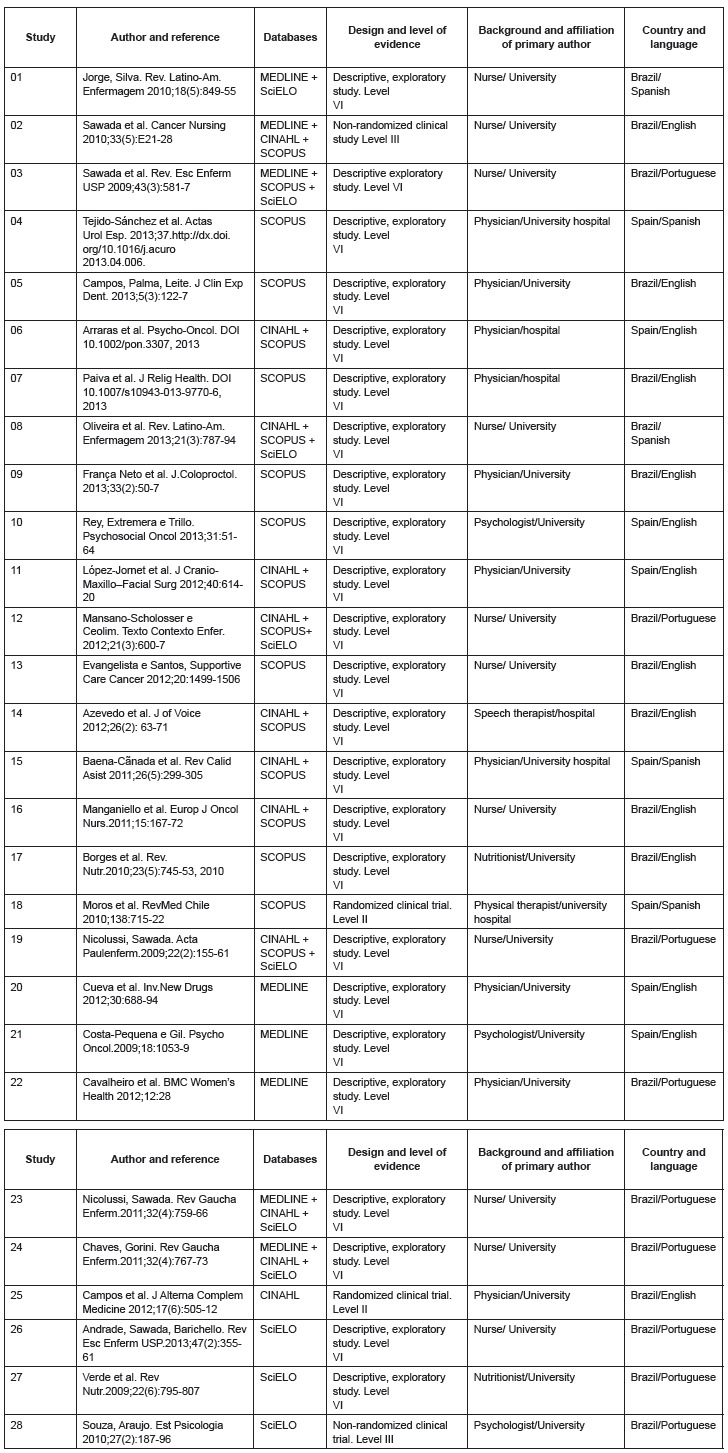
- Description of the studies included in the IR according to author, year of publication, database, study design, and level of evidence, background and affiliation of the primary author, authors' country, and language. Brazil/Spain, 2009-2013

Figure 1 shows the references of the papers, the database in which they were found, the study design and level of evidence, background and affiliation of the primary author, language, and country of origin.

The years of publication of the papers selected indicate an increase in the last two years (2012-2013), with 15 (51.7%) papers. In regard to the country of origin, 71.4% refer to Brazilian studies and only eight (28.5%) papers were conducted in Spain. Of the 28 papers analyzed, the primary authors of 11 (39.2%) papers were nurses; 10 (35.7%) were physicians, three (10.7%) were psychologists, two (7.1%) were nutritionists, one (7.1%) speech therapist, and one (7.1%) physical therapist. Most (22/78.5%) authors were affiliated with universities, three (10.7 %) with university hospitals, and three (10.7%) with other hospitals.

In regard to the methodological characteristics, such as study design and level of evidence, 28 (100%) papers are quantitative studies, while only two (7.1%) are controlled randomized clinical trials with level of evidence II; two (7.1%) are non-randomized clinical studies with level III; and 24 (85.7%) are descriptive and exploratory studies with level VI^(^ 7 ^)^.

In regard to the language, most (14.50%) were written in English, nine (32.2%) in Portuguese and five (17.8 %) in Spanish. Concerning the concept of quality of life, 13 papers (46.4%) defined the concept, while six papers established it as a general concept and seven defined HRQL; the remaining 15 (53.5 %) papers did not define the concept.

All 28 (100%) papers used one or various instruments. Of these, 24 (85.7%) justified their choice, while four (14.2%) did not. In 13 studies, the definition of QoL was coherent with the use of the instrument, while 15 studies did not define the concept of QoL.

The instruments most frequently used to measure QoL were: the European Organization for Research and Treatment of Cancer - EORTC QLQ-C30, with 11 (39.2%) papers and a specific module, EORTC module - BR23 for breast cancer was used in three studies (10.7%) and the Functional Assessment of Cancer Therapy - Breast- FACT-B was used in two (7.1%) studies; EORTC module- HN35 for head and neck cancer was used in one (3.5%) study and EORTC module-CR38 for colorectal cancer was used in one study (3.5%). The World Health Organization Quality of Life Assessment-WHOQOL-100 or WHOQOL-bref were used in five (17.8%) papers each, the Short Form Health Survey-SF-36 in two (7.1%) and the remaining instruments FSFI, FACT-F, EUROQOL 5D 3L,GHQ, UWQOL and SWAL QOL were used only in one study each.

After reading and analyzing the papers, the following categories were established to synthesize knowledge: Category 1 - assessment of QoL in different types of cancer; Category 2 - sociodemographic and clinical factors that influence QoL; and Category 3 - treatments and interventions that improve QoL. Eight papers were included in Category 1 (28.57 %), seven of which were conducted in Brazil and one in Spain; Category 2 included 14 (50%) papers, 10 of which were conducted in Brazil and four in Spain; and Category 3 included six (10.7%) papers, four Brazilian and two Spanish studies. The analysis of papers and the synthesis of knowledge were conducted according to the type of cancer to better understand the results.

**Figure 2:**
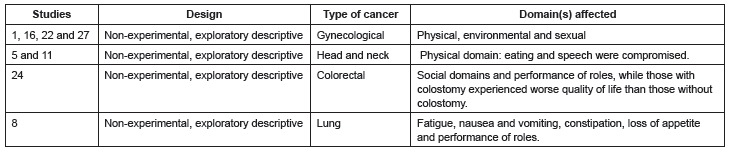
- Category 1: assessment of QoL in different types of cancer

Figure 2 presents the studies included in Category 1, design, type of cancer under study, and QoL domains that were affected.

The four studies addressing gynecological cancer were conducted in Brazil and the sociodemographic and clinical characteristics were similar, such as the mean age of patients, the number older than 50 years old, most were married, and disease was predominantly in stage II, while the participants had attended school for more than nine years.

In the studies addressing head and neck cancer, even though one was conducted in Brazil and the other in Spain, they are similar in regard to the average age of patients, the number older than 50 years old, most being male, and most with basic educational level. In regard to clinical characteristics, in study 5, most characteristics were of laryngeal cancer in stage II, the treatment of which was predominantly radiotherapy and chemotherapy. In study 11, most tumors were located in the oral cavity in stage II and surgical procedures were the primary treatment. 

The study addressing lung cancer was developed in Brazil with the following sociodemographic characterization: average age was 68 years old, most were males, smokers, and were in stage IV, while the predominant protocol of chemotherapy was cisplatin+gemcitabine.

**Figure 3:**
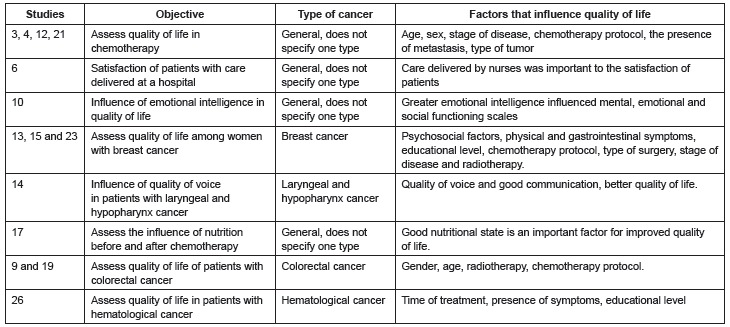
- Category 2: sociodemographic and clinical factors that influence QoL

Figure 3 presents the studies in Category 2, objective and type of cancer and sociodemographic factors that influenced QoL.

Two of the studies 3, 4, 12 and 21 were conducted in Brazil and two in Spain. In the four studies in which colorectal cancer predominated, most patients were male, older than 50 years old on average, married, with primary educational level, most were undergoing chemotherapy. The other study conducted in Spain addressed patients with bladder cancer undergoing adjuvant chemotherapy with the following sociodemographic characteristics: aged 69 years old on average, most were males, cystectomies had been performed within an average of 43 months, and a prevalence of complications with estomy was observed in 61% of the sample.

Study 6 assessed satisfaction of patients with care provided in a cancer hospital in Spain with the following sociodemographic characteristics: aged 60.8 years old on average, most were male individuals (54,6%), basic educational level (55.7%), married (74.4%), type of gastrointestinal tumor (34.1%), and 52.3% had experienced metastasis.

Study 10 was developed in Spain and the sample was predominantly composed of women (87.1%), aged 50.5 years old on average, with time since diagnosis longer than three years (67.74%), secondary educational level (37.7%), married (64.5%), while breast cancer was the most prevalent (70.9%) disease, stage II (62.9%), had undergone surgery and were receiving chemotherapy. 

Studies 13, 15 and 23 addressed breast cancer, two of which were developed in Brazil and one in Spain. Sociodemographic data were similar in regard to average age (50 years old), type of treatment, which was predominantly surgical, and most underwent chemotherapy. In one study, the predominant chemotherapy protocol was anthracycline and 5FU+ cyclophosphamide + doxorubicin was the protocol used in another study; two papers did not specify the protocol. In two studies, the prevailing educational level in the study conducted in Spain was basic education while a bachelor's degree was prevalent in the study conducted in Brazil. In regard to the stage of the disease, stage II was most frequently observed in the study conducted in Spain, while stage I was more frequent in the study conducted in Brazil; the remaining study did not specify the stage of the disease.

Study 14 was developed in Brazil with a sample predominantly composed of men (82.1%) aged 74.5 years old on average, with incomplete primary school (38.1%), while smoking and alcoholism was present in 94% and 82.1% of the sample, respectively.

Study 17 included a sample of Brazilian patients aged 56.6 years old on average, with low socioeconomic status (46.7%), most of whom were women (76.2%) with breast or gynecological cancer (58.7%), stage II (46.5%), in which 77.6% received neoadjuvant chemotherapy and 51.8% underwent surgery before chemotherapy.

Studies 9 and 19 are Brazilian studies, whose samples were mostly composed of male patients (59.1%), aged between 60 and 80 years old (50%), married (50%), retired (63.7%), with basic educational level (59.1%), Catholic (72.7%), while the type of cancer was colon cancer in 72.8% of the cases, 81.8% underwent surgery less than 20 months ago, and the chemotherapy protocol was 5 FU+Leucovorin in 59.1% of the cases.

Study 26 was developed in Brazil with a sample mainly composed of men (56.2%) aged 60 years old or older (56.2%), retired (46.8%), with income from two to four times the minimum wage (50%), incomplete primary education (50%), married (59%), while the type of cancer was leukemia (46.8%) and treatment had started more than four months ago (65.6%).

**Figure 4:**
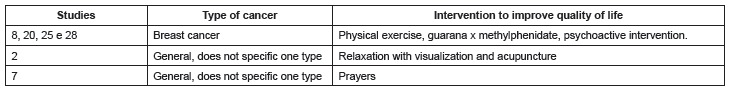
- Category 3: treatments and interventions that improve QoL

Figure 4 presents the studies included in Category 3, type of cancer and interventions that improve QoL.

Two of the studies 8, 20, 25 and 28 were developed in Spain and two in Brazil. The samples of all the studies presented similar sociodemographic and clinical data, average age was older than 50 years old, and stage II was the predominant stage of the disease in one study in Brazil and in another conducted in Spain. The other two studies did not report the stage of disease.

Study 2 assessed relaxation with visualization and acupuncture and was developed in Brazil with a quasi-experimental design in which intervention and control groups assessed at the beginning and end of chemotherapy. The results show that the intervention improved global health/QoL, emotional and social functions and reduced fatigue and loss of appetite. The authors report that small sample was a limitation of the study.

Study 7, a longitudinal descriptive study, assessed individual prayers. A positive correlation was found between individual prayers and QoL among patients receiving palliative care. The type of the instrument used to measure prayers was reported as being a limitation because the form contained one single question due to a lack of valid and reliable instruments, while the design was exploratory descriptive. The sociodemographic and clinical characteristics of studies 2 and 7 were similar, as most were male individuals older than 40 years old, Catholic, and type of gastrointestinal tumor.

## Discussion

In regard to the country of origin, 20 (71.4%) studies were conducted by Brazilian authors and only eight (28.5%) were conducted by Spanish authors. This result may be explained by the fact that nursing graduate programs were only recently implemented and regulated in Spain (Decree 1,393/2007)^(^ 8 ^)^, while in Brazil a Master's Degree in the nursing field was implemented in the 1970s and doctoral programs were implemented in the 1908s^(^ 9 ^)^. This is a topic of great interest in the nursing field, as shown in Figure 1: the primary authors of 11 (39.2%) of the 28 papers under study were nurses. These findings show that QoL of cancer patients undergoing chemotherapy is still a predominant topic among physicians and nurses, professionals who directly provide care to these patients and witness the problems experienced by cancer patients. Another element that becomes apparent is that most scientific production is linked to universities and university hospitals. According to one study^(^ 10 ^)^, the production of knowledge is centered in universities, as these have both qualified personnel and the resources to develop studies.

In regard to the studies' design, most were descriptive exploratory studies with level of evidence VI, showing a lack of controlled randomized clinical studies in this field of knowledge, with stronger levels of evidence for clinical practice. The importance of observational studies, however, should be affirmed, as these studies present some advantages over controlled and randomized clinical trials, such as lower costs, the speed with which they can be conducted, a facility recruiting large samples, and these studies are also mainly used to identify risk factors and prognostic indicators in situations in which controlled randomized clinical trials would be infeasible or unethical^(^ 11 ^)^.

In regard to language, 50% of the studies were written in English and the remaining studies were written either in Portuguese or Spanish. These findings are related to the databases in which the papers were identified. Most papers were found in SCOPUS, MEDLINE and CINAHL, which require papers to be written in English.

In regard to QoL aspects^(^ 12 ^)^, we stress the importance of authors establishing a concept of QoL and this definition should be coherent with the type of instrument used and theoretical framework grounding the instrument and the nature of the study's discussion. The lack of an explicit theoretical model to guide the development of studies addressing quality of life limits the use and generalization of QoL in clinical studies with cancer patients^(^ 13 ^)^.

The instruments most frequently used to assess the QoL of cancer patients undergoing chemotherapy in the papers included in this IR were similar to those found in another IR^(^ 6 ^)^.

Category 1 - assessment of QoL in different types of cancer, gynecological cancers, showed that the QoL factors most frequently affected among patients undergoing chemotherapy were sexual function due to malaise, pain and vomiting that compromised physical well-being, and reduced ability to meet family needs. The patients also experienced weakness, nausea and nuisance caused by the chemotherapy's side effects, which corroborate data of a similar study^(^ 14 ^)^.

All four studies were developed in Brazil; no Spanish study was found addressing the QoL of patients with breast cancer undergoing chemotherapy. This finding confirms the Systematic Review^(^ 15 ^)^ that reports that studies addressing HRQL among patients with breast cancer are seldom conducted in Spain.

In regard to the studies addressing head and neck cancer, the physical domain was the domain most frequently affected, in which patients presented impaired swallowing, speech, compromised teeth, and dry mouth, in addition to pain and fatigue. This information corroborates another international study that also reports swallowing problems, pain, teeth problems, and other comorbidities with onset after treatment for head and neck cancer, compromising the QoL of patients^(^ 16 ^)^.

In these studies, even though one was conducted in Brazil and the other in Spain, sociodemographic data are similar in regard to the average age, and those older than 50 years old. Additionally, most were male, had basic education, and in regard to the clinical characteristics of study 5, most had stage II laryngeal cancer and were predominantly treated with radiotherapy and chemotherapy. In study 11, most had a tumor located in the oral cavity, stage II, and had undergone surgery. These results are similar to those reported in another study^(^ 17 ^)^ that also assessed association of synergism of smoking, alcoholism and depression. Socioeconomic factors are in agreement with systematic reviews^(^ 18 ^)^ of studies conducted in the United States, India, Italy, France, Canada, Germany, Spain, Denmark, England, and Brazil, where an association between head and neck cancer and socioeconomic conditions was more frequently found.

The results found in the study addressing colorectal cancer show that post-operative complications compromised social and professional roles and those who underwent colonoscopy experienced worse QoL than those who did not. These findings are similar to those found in another study^(^ 19 ^)^ which identified the following as risk factors: poor QoL, psychological stress, advanced stage of the disease, and having a stoma.

The study addressing lung cancer reports that after the third cycle of chemotherapy, symptoms such as fatigue, nausea and vomiting, constipation, and loss of appetite become more intense, compromising the functional scale and performance of roles. One study^(^ 20 ^)^ that investigated the symptoms and QoL of patients with lung cancer report that fatigue was the most frequently experienced among these patients and detected that this symptom influenced the functional performance of patients, worsening their QoL.

Assessing and improving the quality of life of patients during and after cancer treatment is essential and a factor that elicits recognition of good practices in oncological services worldwide; many services are recognized and awarded because of this aspect ^(21)^.

In Category 2, the four studies addressing QoL among patients undergoing chemotherapy shows that age, being a woman, stage of the disease, and chemotherapy protocols, and adjuvant chemotherapy cause more symptoms and worsen QoL of patients. Various Brazilian and international studies have shown the influence of these factors in the HRQL of patients undergoing chemotherapy^(^ 22 ^-^ 24 ^)^.

The study conducted in Spain that assessed association of satisfaction with a service and QoL reports that the domains related to care provided by physicians were lower, while those related to nurses were higher. One study^(^ 25 ^)^ developed in Brazil that verified the interaction of nurses with patients undergoing chemotherapy also reports that nursing care is based on principles inherent to human relationships, such as friendship, affection, attention, tolerance, and solidarity. It stresses that nurses' actions combine technical and human attributes considering life as an essential ethical value in respect to human dignity, as the basis for interactions in care delivery, which shows the need for humanized interactions among the multidisciplinary staff to help patients with cancer overcome their situation with good quality of life.

Influence of quality of voice and swallowing among patients with head and neck cancer was demonstrated in another study^(^ 26 ^)^ that was conducted in Brazil.

The relationship between emotional intelligence and physical and mental health was demonstrated in a review^(^ 27 ^)^ that found that high levels of emotional intelligence are related to improved mental health, while low emotional intelligence is related to certain emotional disorders, which was also reported by the paper found in this IR.

Studies regarding breast cancer report that the factors that influence QoL were psycho-emotional, physical, gastrointestinal, age, level of education, type of chemotherapy, surgical treatment, stage of the disease, body image, and level of anxiety and depression. Many studies report these factors^(^ 14 ^-^ 15 ^)^.

Malignant neoplasia cause metabolic alterations in patients and have been classified as a state of nutritional risk. Malnutrition among adult cancer patients ranges from 40% to 80%, while it ranges from 6% to 50% among child patients. It is directly associated with worsened QoL due to a lower response to specific treatment^(^ 28 ^)^. This information was found in a paper included in this review, where nutritional state was a positive factor for improved QoL.

The studies that assessed colorectal cancer report that sex influences QoL, where women present more deficits and symptoms than men, while patients younger than 50 years old experience fewer problems with urination. Radiotherapy influenced symptoms such as pain, insomnia, and the performance of roles. The chemotherapy protocol influenced the cognitive functions and symptoms of pain and loss of appetite. In another study^(^ 19 ^)^ patients with colorectal cancer were prospectively assessed and no significant difference was found in regard to age, marital status, educational level, or stage of tumor in regard to HRQL. The study reports that patients who underwent surgery and adjuvant chemotherapy presented improved physical and functional well-being compared to those who had only undergone surgery, while the most optimistic patients presented association with all the HRQL domains with the exception of social well-being.

A study addressing patients with hematological cancer detected that the presence of symptoms influences physical, cognitive and social functions. Time of treatment influenced a greater presence of symptoms, such as nausea and vomiting and loss of appetite. Years of schooling influenced the social function scale and financial difficulties, while those with fewer years of schooling experienced more financial hardships. One international study^(^ 29 ^)^, addressing 1,482 patients with leukemia, found that the following factors were related to HRQL: more advanced age, fatigue, severity of comorbidities, and current conditions of treatment, have a more profound impact on HRQL in all the stages of the disease. The effects of leukemia on QoL seem to be different than other types of cancer, with a greater impact on the emotional dimension.

Category 3 - treatments and interventions that improve QoL was composed of six studies, two of which had experimental designs, two had quasi-experimental designs while two studies had longitudinal descriptive designs. Four studies addressed populations of women with breast cancer and tested physical exercise, the use of methylphenidate, guarana, and a psycho-educational intervention. In two studies, one assessed the relaxation technique with visualization and acupuncture and the other assessed prayers among patients with cancer undergoing chemotherapy. All the interventions were effective in the improvement of symptoms such as fatigue and stress, which resulted in improved QoL. All the interventions, except the use of methylphenidate, are complementary practices.

The use of complementary and alternative medicine has increased in recent years. The Systematic Review^(^ 30 ^)^ showed that the profile of patients is as follows: adults, aged between 30 and 59 years old, female, high educational level, high family income, and with advanced disease. The participants were part of some religious tradition, ethnically influencing the type of complementary therapy that was adopted. The complementary therapies most frequently used were homeopathy, Ayurveda medicine, Chinese traditional medicine, herbal medicine, psychological therapies, spiritual therapies, support groups, relaxation and meditation, diets, and reflexology. Data are similar to those found in a study^(^ 31 ^)^ that analyzed complementary and alternative medicine in European countries and found that one third of patients with cancer seek these treatments, mostly young women with a high level of education. The most common therapies include herbal medicine, homeopathy, and spiritual therapies.

A study that assessed physical exercise among women with cancer reports that the benefits are reduced fatigue. The Systematic Review^(^ 32 ^)^ reports that the effects of resistance training on the QoL of patients with cancer also showed that training programs benefit patients and, consequently, improve QoL. Note, however, that the type, intensity, and quantity of exercise should be appropriate to patients' conditions. 

Body/mind techniques have gained attention in the treatment of cancer patients. One study^(^ 33 ^)^ emphasizes discoveries regarding the biochemical interaction that takes place among the neurological, endocrine and immunological systems, together with emotional modulation of response to stress. Relaxation and creative visualization and the relationship between health and spirituality are discussed as useful tools to acquire balance between stress and relaxation.

Acupuncture has also been greatly used in the treatment of chemotherapy side effects, such as nausea and vomiting, fatigue, loss of appetite, insomnia, pain, constipation, and depression and anxiety, improving the QoL of patients with cancer^(^ 34 ^)^. The papers analyzed in this review employed relaxation with guided visualization and prayers, showing that cancer patients undergoing chemotherapy were benefited.

The review^(^ 35 ^)^ on fatigue among cancer patients found that pharmacological treatment with methylphenidate and dexmethylphenidate and the use of guarana (*Paulinia cupana*) among patients with severe fatigue were also beneficial. The studies in this IR corroborate this finding.

## Conclusions

This IR gathered 28 studies addressing HRQL of Brazilian and Spanish cancer patients undergoing chemotherapy. All the papers were quantitative: 24 (86%) were descriptive cross-sectional studies with evidence level VI; two (7%) were experimental studies with evidence level II; and two were quasi-experimental studies with evidence level III, showing a poor level of evidence overall. The instrument most frequently used was EORTC QLQ-C30, while most studies were conducted in Brazil, whose authors were predominantly physicians and nurses affiliated with universities. 

Chemotherapy harms HRQL domains. The most influential factors include: age, sex, type of chemotherapy protocol, type of surgery, stage of the disease, level of education, and level of emotional intelligence. Despite the low level of evidence, the complementary therapies included: acupuncture, guided visualization, prayers, and exercise, which were effective in reducing chemotherapy side effects, while methylphenidate and *guarana* reduced fatigue in patients undergoing chemotherapy. These results can help the care planning of Brazilian and Spanish cancer patients undergoing chemotherapy, contributing to clinical practice.

Note that the delimitation of studies conducted in Brazil and Spain is a limitation of this study; however, as previously justified, this study is part of a larger project between the two countries. Another limitation is the reduced number of papers published in Spain in comparison to Brazil, which hindered a deeper analysis between the two populations.
